# Exploring and linking biomedical resources through multidimensional semantic spaces

**DOI:** 10.1186/1471-2105-13-S1-S6

**Published:** 2012-01-25

**Authors:** Rafael Berlanga, Ernesto Jiménez-Ruiz, Victoria Nebot

**Affiliations:** 1 Departamento de Lenguajes y Sistemas Informáticos, Universitat Jaume I, Campus Riu Sec s/n, E-12071 Castellón, Spain; 2 Department of Computer Science, University of Oxford, Wolfson Building, Parks Road, Oxford OX1 3QD, UK

## Abstract

**Background:**

The semantic integration of biomedical resources is still a challenging issue which is required for effective information processing and data analysis. The availability of comprehensive knowledge resources such as biomedical ontologies and integrated thesauri greatly facilitates this integration effort by means of semantic annotation, which allows disparate data formats and contents to be expressed under a common semantic space. In this paper, we propose a multidimensional representation for such a semantic space, where dimensions regard the different perspectives in biomedical research (e.g., population, disease, anatomy and protein/genes).

**Results:**

This paper presents a novel method for building multidimensional semantic spaces from semantically annotated biomedical data collections. This method consists of two main processes: knowledge and data normalization. The former one arranges the concepts provided by a reference knowledge resource (e.g., biomedical ontologies and thesauri) into a set of hierarchical dimensions for analysis purposes. The latter one reduces the annotation set associated to each collection item into a set of points of the multidimensional space. Additionally, we have developed a visual tool, called 3D-Browser, which implements OLAP-like operators over the generated multidimensional space. The method and the tool have been tested and evaluated in the context of the Health-e-Child (HeC) project. Automatic semantic annotation was applied to tag three collections of abstracts taken from PubMed, one for each target disease of the project, the Uniprot database, and the HeC patient record database. We adopted the UMLS Meta-thesaurus 2010AA as the reference knowledge resource.

**Conclusions:**

Current knowledge resources and semantic-aware technology make possible the integration of biomedical resources. Such an integration is performed through semantic annotation of the intended biomedical data resources. This paper shows how these annotations can be exploited for integration, exploration, and analysis tasks. Results over a real scenario demonstrate the viability and usefulness of the approach, as well as the quality of the generated multidimensional semantic spaces.

## Background

The ever increasing volume of web resources as well as generated data from automated applications is challenging current approaches for biomedical information processing and analysis. One current trend is to build semantic spaces where biomedical data and knowledge resources can be mapped in order to ease their exploration and integration. Semantic spaces are usually defined in terms of widely accepted knowledge resources (e.g. thesauri and domain ontologies), and they are populated by applying (semi)automatic semantic annotation processes. This is the result of a decade of integration initiatives aimed at inter-linking and merging publicly available biomedical databases (see [1] for a recent review). Most of these initiatives have followed a warehousing approach, where existing data are loaded into a central store under a common schema (e.g., BioMART [2], EcoCyC [3], and Ondex [4]). Recently, with the emergence of the Web of Data [5], this integration effort is being performed in the context of the Semantic Web under standard formats like RDF [6] and OWL [7].

Additionally to these integration projects, literature based discovery (LBD) [8] has aimed at inferring implicit knowledge by mining scientific papers. LBD approaches also take profit from knowledge resources in order to identify biomedical entities in the texts as well as their associations (see the recent reviews in [9], [10] and [11]).

Visualization tools play a very relevant role in integration and LBD projects. This is because summarized visual information is required for analyzing the huge amount of data involved in these projects. In this context, visual inference has shown useful in many biomedical research projects [11]. The main visual representation adopted in these projects is the conceptual map, where entities (or concepts) and their asserted or inferred associations are visualized as a graph. Cytoscape [12] and Ondex [4] are the main representatives for integration projects, and Telemakus [13] and LitLinker [14] are examples of visualization tools for LBD.

The main limitation of current visualization tools is that they have been developed as stand-alone applications, requiring all the data to be locally loaded and processed. This makes it unfeasible to deal with very large data collections as well as to visualize the information in portable small devices such as mobile phones and tablets. Clearly, a web-based architecture is more appropriate for performing visual analysis tasks over huge amounts of integrated data. However, as far as we know, there are no web-based interfaces providing rich and dynamic visualizations for analyzing biomedical data. Instead, web services are designed to provide discovered knowledge and biomedical data in plain formats (e.g., [15-17]). Our approach proposes the use of On-Line Analytical Processing (OLAP) techniques [18] to integrate and visualize large collections of biomedical data from conventional web browsers. OLAP technology has shown very successful for analyzing summarized data from different perspectives (dimensions) and detail levels (categories). Part of this success is due to its simplicity in data structures and its efficiency performing data summaries. In a typical OLAP architecture, data are integrated and pre-processed in the back-end (e.g., a data warehouse), so that the amount of data users receive in the client side (e.g., a web browser) is dramatically reduced. Moreover, OLAP tools provide a series of operators that allow users to interact with the summarized information as well as to get more detailed information of those parts she wishes to explore. All these features overcome the previously mentioned limitations of current biomedical visualization tools. In this paper, we propose a novel method for building multidimensional semantic spaces from semantically annotated biomedical databases. The main aims of these semantic spaces are: to provide a summarized view of the data sources, to find interesting associations between concepts present in the collection, and to visualize the collection contents for exploration purposes. As in most of the reviewed visualization tools, conceptual maps have been also adopted in our approach to visualize the integrated data. However, our conceptual maps have three main distinctive features: (1) concepts are arranged into a set of pre-defined biomedical research perspectives, (2) the visualization is oriented to perform OLAP-based operations, and (3) the visualization is rendered in a 3D scenario. The first feature enables a more structured visualization, where associations (called bridges) must involve entities of different levels (e.g., *Disease *versus *Protein*). The second feature is related to the interactivity of the user with the visualized data. Finally, the latter feature allows a better use of the space to allocate as much data as possible. It must be pointed out that conceptual maps are dynamically built from the web browser according to the users requirements, by selecting the appropriate levels to be visualized. The current implementation of this method is publicly available in [19] for testing purposes.

The paper is organized as follows. First, the Methods Section is devoted to introduce the methodological aspects of our approach. First, we describe the normalization formalism to represent both the knowledge resources and the target data collections, and the OLAP-like operators defined over the normalized representation (i.e., multidimensional space). Afterwards, in the Results Section, we describe some use examples to show the functionality of the implemented prototype, and the experiments carried out to evaluate the quality of the visualized data. Finally, we give some conclusions and future work.

## Methods

OLAP (On-line Analytical Processing) [20] tools were introduced to ease information analysis and navigation from large amounts of transactional data. OLAP systems rely on multidimensional data models, which are based on the fact/dimension dichotomy. Data are represented as facts (i.e., subject of analysis), while dimensions contain a hierarchy of levels, which provide different granularities to aggregate the data. One fact and several dimensions to analyze it give rise to what is known as the data cube. Common operations include: *slice*, which performs a selection on one dimension of the given cube resulting in a sub-cube, *dice*, which performs a selection on two or more dimensions, *drill-down*, which navigates among levels of data ranging from the most summarized (up) to the most detailed (down), *roll-up*, which is the inverse of drill-down, *pivot*, which rotates the data to provide an alternative presentation, and *drill-through*, which accesses the detailed data that is involved in the summary.

Since multidimensional models provide a friendly, easy-to-understand and intuitive visualization of data for non-expert end-users, we have borrowed the previous concepts and operations to apply them to the proposed conceptual maps.

This section is devoted to present the necessary methods to generate and manage multidimensional semantic spaces.

### Overview of the architecture

Unlike other visual integration approaches like Ondex [4], in our approach knowledge resources (KRs) are distinguished from data resources (DRs). KRs are well-structured databases consisting of concepts and their relationships (e.g., GO and UMLS), whereas DRs are any kind of biomedical database to be integrated under some reference KR. DRs are usually semi-structured and text-rich (e.g., PubMed abstracts, patient records, the OMIM database [21], Uniprot, and so on). For the sake of simplicity, we assume that a DR consists of a collection of uniquely identified items, whose contents can present arbitrary structures (e.g., relational, XML, plain text, etc.)

**Figure 1:**
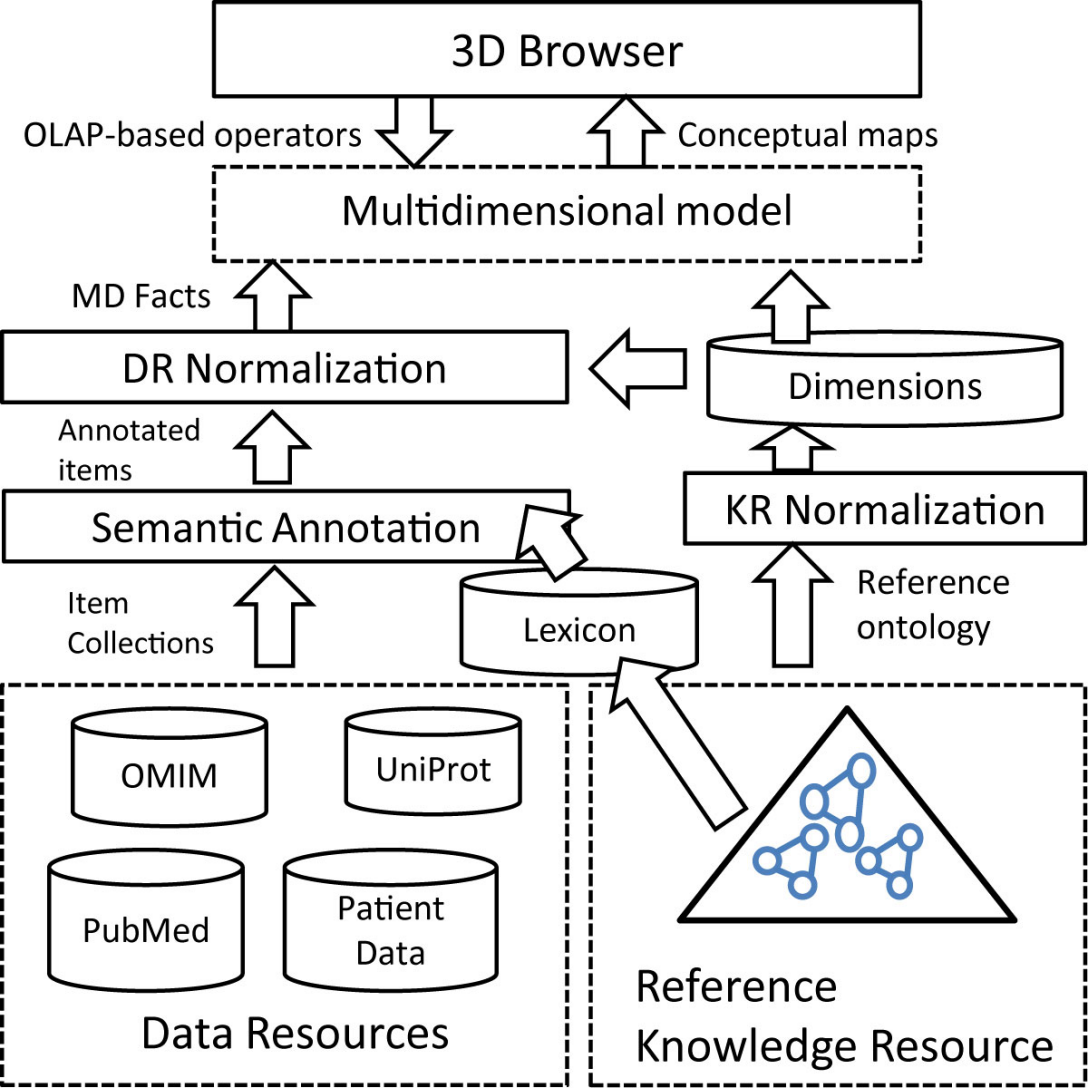
**Overview of the proposed method**. Proposed method for integrating and normalizing data resources (DRs) and knowledge resources (KRs).

Figure 1 summarizes the proposed method for generating browsable analytical semantic spaces. As a first step, the DRs and the reference KR must be normalized. KR normalization consists in organizing the KR concepts into a well-structured multidimensional schema, whereas DR normalization consists in representing the DR's items under this schema. Multidimensional schemas are set up in terms of a series of predefined *dimensions *which roughly represent *semantic groups*. For example, in systems biology, a semantic group can comprise entity types playing a specific role, for example *Gene*, *mRNA*, *Polypeptide*, *Physiological_Function *and *Metabolite*. In the biomedical domain, examples of dimensions are *Population*, *Disease*, *Organ*, *Tissue*, and so on.

DR normalization is performed in two steps: (1) semantic annotation of the DR collection items with concepts from the reference KR, and (2) normalization of each collection item to the multidimensional schema derived from the normalized KR. The subsequent sections are devoted to describe in detail these normalization processes as well as the generation of semantic bridges.

### Semantic annotation

During the last years, we have witnessed a great interest in massively annotating biomedical scientific literature. Most of the current annotators rely on well-known lexical/ontological resources such as MeSH, Uniprot, UMLS and so on. These knowledge resources usually provide both the lexical variants for each inventoried *concept *and the concept taxonomies.

In our work, the knowledge resource used for generating semantic annotations is called *reference ontology*, denoted with *O*. The lexical variants associated to each ontology concept *c *are denoted with *lex*(*c*), which is a list of strings. The taxonomic relations between two concepts *a *and *b *are represented as *a *≼ *b*. A semantic annotation of a text chunk *T *is the task of identifying the most specific concepts in *O *such that they are more likely to represent the meaning of *T*.

Most semantic annotation systems are dictionary look-up approaches, that is, they rely on the lexicon provided by the ontology in order to map text spans to concept lexical variants. Some popular annotation systems in the biomedical domain are Whatizit [22] and MetaMap [23], which rely on GO and UMLS resources respectively.

It must be pointed out that MetaMap has been widely used in literature-based discovery to identify biomedical entities in the mined texts. However, this kind of tool does not scale well for very large collections. To overcome this limitation, annotations are restricted to a few entity types or to the MeSH controlled vocabulary. Another limitation of this tool is that it is not extensible with new concepts and lexical variants coming from other KRs.

In our work we have adapted an annotation system called Concept Retrieval (CR) [24], which scales well over large collections as well as large KRs. Moreover, this system can easily include any kind of KR and deal with merged KR lexicons. This annotation system was tested in the two CALBC competitions [25,26] over a collection of 864 thousand PubMed abstracts about immunology [27], which is annotated in less than 8 hours.

The idea behind the CR system consists in ranking the lexical strings of the lexicon with respect to each target text chunk *T *by applying the following formula:

$$rank\left(S_{i},T\right)=\frac{idf\left(S_{i}\right)-idf\left(S_{i}-T\right)}{idf\left(S_{i}\right)}\cdot \frac{\left|S_{i}\cap T\right|}{ambiguity\left(S_{i}\right)}$$

Concept strings *S*_*i *_and text chunks *T *are represented as bags of words. The function *idf*(*S*) represents the amount of information contained in the concept string *S*, which is estimated as follows:

$$idf\left(S\right)=-\underset{w\in S}{\sum }p\left(w|Background\right)$$

In the current implementation we use the whole Wikipedia as the *Background *collection for estimating word probabilities. Finally, the function *ambiguity*(*S*) returns the number of concepts that have *S *as lexical variant. To sum up, the formula above promotes those strings with high information amount, long matches, and low ambiguity degree.

**Figure 2:**
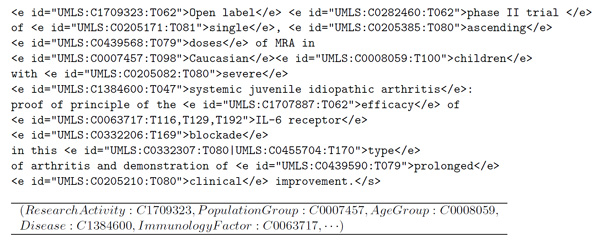
**Semantic annotation example**. This figure presents an example of a semantically tagged text. Semantic annotations are expressed in IeXML format, where the cross-references to the reference ontology are included in the *id *attribute of the XML *e *tags. A cross-reference consists of three parts separated by the colon character: the source (e.g., UMLS), the concept identifier (e.g., C0007457), and the semantic type (e.g., T098). The bottom side of the figure shows the multidimensional fact generated from this tagged text.

The final annotation is generated by taking the top ranked concepts that cover as many words as possible from the text chunk *T*. As an example, Figure 2 shows the semantic annotations generated by the CR annotator.

### Knowledge resource normalization

In order to build semantic spaces for analyzing document collections, the reference ontology *O *associated with the knowledge resource has to be normalized into a well-structured multidimensional schema. The main issue to be addressed in this process is to manage the highly irregular structures of the KR taxonomies. With this issue in mind, the KR normalization is performed as follows:

• First a set of dimensions are defined, (*D*_1_, ... *D*_*n*_), which represent a partition of the concepts in the domain ontology. Each dimension *D*_*i *_represents a different semantic space (e.g. semantic types or vertical levels), and cannot share any common sub-concept with the other dimensions.

• Each dimension *D*_*i *_can define a set of categories or levels $L_{j}^{i}$, which forms in turn a partition over *D*_*i *_but with the following constraints: (1) there cannot be two concepts *c *and *d *in $L_{j}^{i}$ such that either *c *≼ *d *or *d *≼ *c*, and (2) all the concepts in $L_{j}^{i}$ have a common super-concept that belongs to *D*_*i*_. By imposing these constraints we ensure summarizability and good OLAP properties for the generated dimensions hierarchies.

• In order to efficiently build the dimensions hierarchies from the reference ontology *O *with such constraints, we index the taxonomic relationships using intervals as presented in [28]. This way, every concept of *O *has associated two sets of intervals corresponding to its ancestors ($L^{+}$) and descendants ($L^{-}$) in the ontology. By using an interval's algebra over this representation, we are able to query about the taxonomic relationships between concepts as well as to compute common ancestors and descendants. For example, let *c *= ([4, 9]^-^, [9, 10]^+^) and *d *= ([7, 7]^-^, [3, 3]^+^, [6, 11]^+^) be two indexed concepts. We infer *d *≼ *c *because [7, 7]^- ^⊆ [4, 9]^-^. Similarly, we can obtain common ancestors of *c *and *d *by intersecting the intervals of the ancestors space, ([9, 10]^+^) ∩ ([3, 3]^+^, [6, 11]^+^).

In this way, we can automatically build each dimension *D*_*i *_with the ontology fragment obtained with the signature formed by all the concepts identified in the collection (through semantic annotation) and that belong to some semantic group representing the dimension. To obtain the categories of a dimension *D*_*i*_, we take into consideration the taxonomic relationships in the fragment and the previous restrictions over dimensions and their categories.

### Data resource normalization

After semantic annotation, each item of the target collection Col has associated a list of concepts from the reference ontology *O*. However, these annotation sets are not suited for multidimensional analysis, and therefore a normalization process similar to that applied to the ontology must be performed. The main goal of this normalization is to represent the semantic annotations within the normalized multidimensional space described in the previous section. Thus, each item d∈Col is represented as the multidimensional fact:

$$fact\left(d\right)=\left(D_{1}=c_{1},\cdots \;,D_{n}=c_{n}\right)$$

where *c*_*i *_(0 ≤ *i *≤ *n*) is either a concept from the dimension *D*_*i *_or the *null *value.

As a semantic annotator can tag more than one concept of the same dimension, the normalization process basically consists in selecting the most relevant concepts for each dimension. One issue to take into account in this process is the presence of ambiguous annotations, that is, when more than one concept is assigned to the same text chunk. We say that two concepts are in conflict when they are included in some ambiguous annotation. For example, the string "follow-up" is annotated with two concepts *C*1704685 (report) and *C*1522577 (activity), and therefore they are in conflict.

The selection of relevant and right concepts for each document *d *is performed through a reduction process based on a concept affinity matrix *M*^*d *^of size *N*_*c *_× *N*_*c*_, where *N*_*c *_is the number of distinct concepts present in the annotations of *d*. The idea behind this matrix is to capture the affinity of the concepts associated to each item, so that the more similar a concept is with its neighbors the more relevant it is. The affinity matrix is calculated as the linear combination of the following matrices:

$$M^{d}=M_{isa}+M_{ancs}+M_{R}+M_{sents}^{d}$$

These matrices are defined as follows:

• *M*_*isa*_(*i*, *j*) = 1 iff *c*_*i *_≼ *c*_*j *_in the reference ontology *O*, that is, two concepts are similar if one is a sub-concept of the other,

• *M*_*anc*_(*i*, *j*) = |*common_ancestors*(*c*_*i*_, *c*_*j*_)|/*γ*, being *γ *a parameter that depends on the taxonomy depth, that is, the more ancestors two concepts share the more similar they are,

• *M*_*R*_(*i*, *j*) = 1 iff ∃*R*(*c*_*i*_, *c*_*j*_) ∈ *O*, that is, two concepts related to each other through some relation *R *are deemed similar,

• $M_{sents}^{d}\left(i,j\right)=1$, if *c*_*i *_and *c*_*j *_co-occur in a same sentence of the document *d *and they are not in conflict.

The affinity matrix can be used in many ways to rank the annotation concepts of an item. For example, we can use any centrality-based algorithm to obtain the concept ranking. However, our aim is not only to get the concepts ranking but also to solve the ambiguities produced by the annotation system. For this reason, we require a classification framework able to perform both tasks. The chosen framework is that presented in [29], which is called regularization framework, and which models the classification as an optimization problem over graphs expressed in matrix notation as follows:

(1) $$R^{d}={\left(\left(1-\alpha \right)\cdot {\left(I-\alpha S^{d}\right)}^{-1}\cdot Y^{T}\right)}^{T}$$

Here, *R *is the calculated vector representing the rank of concepts present in the annotations of the collection item *d*, denoted *C*^*d*^. This ranking is obtained by finding an optimal smoothed function that best fits a given vector *Y*, which is achieved by applying the laplacian operator over the affinity matrix *M*^*d *^as follows:

$$S^{d}=D^{-1∕2}\cdot M^{d}\cdot D^{-1∕2}$$

The parameter *α *is directly related to the smoothness of the approximation function (we set it to *α *= 0.9). For disambiguation purposes, each ambiguous annotation *A *⊆ *C*^*d *^is associated to a vector *Y *where *Y*_*i *_= 0 if *c*_*i *_∈ *A *and 1 otherwise. After computing *R*^*d *^with this vector, we can reject all the ambiguous concepts in *A *whose score is lower than the maximum in *R*^*d*^. Rejected concepts imply a reduction in the matrix *M*^*d*^, and we can apply again the disambiguation process until no more concepts are rejected. For ranking purposes, the vector *Y *consists of the frequencies of each concept within the item *d*. Once the rank *R*^*d *^is obtained, the normalization process selects the top-scored concepts of each dimension to represent the *d*'s fact. As an example, Figure 2 shows the resulting fact for the example tagged text. Since collection items are mapped to a set of disjoint dimension concepts in the resulting conceptual map, the relevance of each concept can be measured in terms of the items that support it. The relevance of a concept *c *∈ *O *can be calculated by aggregating the relevance of its sub-concepts w.r.t each specific collection. Formally,

$$Rel_{Col}\left(c,D_{i}\right)=\Gamma_{\forall c^{\prime }\in descendants\left(D_{i},c\right)}score_{Col}\left(c^{\prime }\right)$$

where Γ is an aggregation function (e.g., sum, avg, and so on) and *score *is the function that is evaluated against the collection. The simplest scoring function is the number of hits, namely:

$$score_{Col}\left(c\right)=hits_{Col}\left(c^{\prime }\right)=count\left(\left\{d|d\in Col,fact\left(d\right)\left[D_{i}\right]=c^{\prime }\right\}\right)$$

Alternatively, the scoring function can take into account the relevance of each concept in the items it appears. Thus, we can aggregate the relevance scores estimated to select concept facts as follows:

$$score_{Col}\left(c\right)=\underset{d\in Col,\exists i,fact\left(d\right)\left[D_{i}\right]=c}{\sum }R^{d}\left[c\right]$$

### Semantic bridges

**Figure 3:**
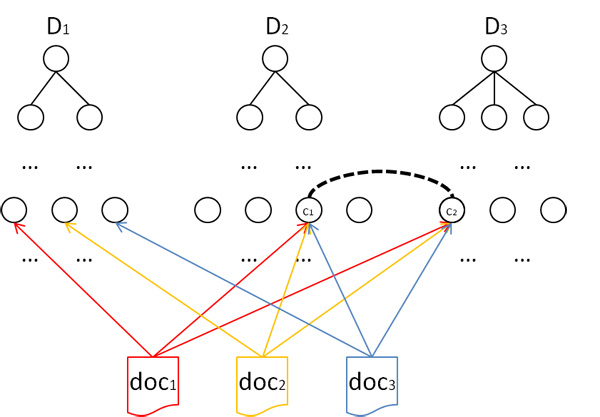
**Graphical representation of a semantic bridge**. *D*_1_, *D*_2 _and *D*_3 _are the dimension hierarchies. The normalization of each document *doc*_*i *_into a fact is represented by the document pointing to the selected concept of each dimension. The semantic bridge between *c*_1 _and *c*_2 _is derived from the associations found in *doc*_1_, *doc*_2 _and *doc*_3_. This setting indicates a strong association between concepts *c*_1 _and *c*_2_, which is supported by documents *doc*_1_, *doc*_2 _and *doc*_3_.

A semantic bridge is any interesting association between concepts of two different dimensions. Interesting associations can be derived from the facts extracted from the target data sources. Figure 3 illustrates the notion of semantic bridge by means of an example. Next, semantic bridges are formally introduced. Given two dimension levels $L_{n}^{i}$ and $L_{m}^{j}$, belonging to dimensions *D*_*i *_and *D*_*j *_(*i *≠ *j*) respectively, the following cube stores the aggregated contingency tables necessary for correlation analysis:

$$CUBE_{Col}\left(L_{n}^{i},L_{m}^{j}\right)=\left\{\left(c_{i},c_{j},n_{i,j},n_{i},n_{j}\right)|c_{i}\in L_{n}^{i}\wedge c_{j}\in L_{m}^{j}\right\}$$

Here *n*_*i,j *_measures the number of objects in the collection where *c*_*i *_and *c*_*j *_co-occur, *n*_*i *_is the number of objects where *c*_*i *_occurs, and *n*_*j *_is the number of objects where *c*_*j *_occurs. Notice that *n*_*i *_and *n*_*j *_are calculated in a similar way as concept relevance. The contingency table for each pair (*c*_*i*_, *c*_*j*_) is calculated as shown in Table 1.

**Table 1:** Contingency table for scoring bridges

	***c***_***i***_	${\bar{c}}_{i}$
*c*_*j*_	*n*_*i,j*_	*n*_*j *_-*n*_*i,j*_
${\bar{c}}_{j}$	*n*_*i *_*-n*_*i,j*_	*N*_*Col *_*-n*_*i *_*-n*_*j*_

The contingency table accounts for the number of observations of present (i.e., *c*_*i *_and *c*_*j*_) and/or absent (i.e., ${\bar{c}}_{i}$ and ${\bar{c}}_{j}$) concepts in facts.

The measures *n*_*i,j*_, *n*_*i *_and *n*_*j *_are calculated as follows:

$$\begin{array}{cc} n_{i,j} & =\left|\left\{d|d\in Col\wedge fact\left(d\right)\left[D_{i}\right]≼c_{i}\wedge fact\left(d\right)\left[D_{j}\right]≼c_{j}\right\}\right|\\ n_{i} & =\left|\left\{d|d\in Col\wedge fact\left(d\right)\left[D_{i}\right]≼c_{i}\right\}\right|\\ n_{j} & =\left|\left\{d|d\in Col\wedge fact\left(d\right)\left[D_{j}\right]≼c_{j}\right\}\right| \end{array}$$

Semantic bridges can be now calculated from contingency tables by defining a scoring function *ϕ*(*c*_*i*_, *c*_*j*_). In this way, bridges will be those concept associations whose scores are greater than a specified threshold *δ*:

$$Bridges_{Col}^{\phi }\left(L_{i},L_{j}\right)=\left\{\left(c_{i},c_{j},\phi \left(c_{i},c_{j}\right)\right)|\phi \left(c_{i},c_{j}\right)>\delta \right\}$$

In association analysis [30], scoring functions are used to measure the kind of correlation one can find between several items. Traditionally, the confidence of the rule *c*_*i *_→ *c*_*j *_has been used, which is defined as:

$$\phi \left(c_{i},c_{j}\right)=conf\left(c_{i},c_{j}\right)=\frac{n_{i,j}}{n_{i}}$$

However, this measure presents some limitations. For example, it is not able to distinguish between positive and negative correlations. Thus, other measures like the interest factor can be used instead:

$$\phi \left(c_{i},c_{j}\right)=IF\left(c_{i},c_{j}\right)=\frac{n_{i,j}\cdot N}{n_{i}\cdot n_{j}}$$

As in a collection we can find many kinds of correlations, we use a comprehensive set of well-known interestingness measures to find all the interesting bridges between two levels. Examples of these measures are log likelihood ratio, mutual information and F1-measure. More information about this kind of measures can be found in [30].

One special kind of bridges are those that maximize some interestingness measure for each pair of concepts of the two compared levels. We call these bridges *δ*-maximum interesting pairs. These bridges will serve us for evaluating the quality of the generated bridges for different collections.

From the implementation point of view, bridges can be either pre-calculated and stored in the back-end or generated on the fly. In the former case, the pre-calculation of all the bridges for all the level combinations can result in very large data sets. In the latter case, although it makes the browser slightly slower, the calculation is only performed when drilling-down a concept, which usually involves a few new concepts, and therefore it is efficient to calculate their bridges w.r.t. the visualized concepts.

### Operations over the conceptual map

Our main aim is to build a browsable representation of the semantic spaces defined previously. For this purpose, we define the conceptual map as a sequence of different layers that correspond to different dimensions expressed at some detail level (category). In this map, concepts are visualized as balls, which are placed within their corresponding layer with a size proportional to their relevance w.r.t. the target collection. Concept bridges (or conceptual associations) are visualized as links between concepts of adjacent layers. Conceptual maps are built from the normalized conceptual representation described previously. Table 2 summarizes the main operations over conceptual maps.

**Table 2:** Operations over the conceptual map

Operation	Back-end action	Client-side action
*drillDown* (*D*_*i*_, *c*)	Retrieve the children of *c *in dimension *D*_*i*_	Visualization of direct sub-concepts of *c *and the bridges involved by them.

*contains *(*D*_*i*_, *c*, *kywds*)	Check if *c *has some sub-concept *c' *mathing *kywds*	If true, the concept *c *is visualized with a different color.

*drillThrough *(*D*_*i*_, *c*)	Retrieve the ranking of items *d *annotated with *c *by *rel*(*d*, *c*) = *R*^*d*^[*c*]	Visualization in a tab (one for each indexed collection) of the ranked list of objects with their metadata and cross-references.

*drillThrough* (*D*_*i*_, *b*)	Get the ranking of items *d *supporting the selected bridge *b *= (*c*_1_, *c*_2_) by *rel*(*d*, *b*) = *rel*(*d*, *c*_1_) ·*rel*(*d*, *c*_2_)	Visualization in a tab of the ranked list of objects with their metadata and cross-references.

*conceptRemoval* (*D*_*i*_, *c*)	None	Removal of *c *from *D*_*i *_and all its bridges.

*conceptSelection* (*D*_*i*_, *c*)	None	Removal of all other concepts and their bridges in *D*_*i *_except for *c*.

This table describes the main operations over a conceptual map and the actions they involve in both the back-end (e.g., database server) and the client-side (e.g., the browser).

## Results

The work presented in this paper has been mainly developed in the context of the European Health-e-Child (HeC) integrated project [31,32]. HeC aimed to develop an integrated health care platform to allow clinicians to access, analyze, evaluate, enhance and exchange integrated biomedical information focused on three pediatric domains: heart disorders, inflammatory disorders and brain tumors. The biomedical information sources covered six distinct levels of granularity, also called vertical levels, classified as molecular (e.g., genomic and proteomic data), cellular (e.g., results of blood tests), tissue (e.g., synovial fluid tests), organ (e.g., affected joints, heart description), individual (e.g., examinations, treatments), and population (e.g., epidemiological studies). To represent these levels and annotate data resources, we have selected the Unified Medical Language System Metathesaurus (UMLS) [33] as the reference knowledge resource, which constitutes the main multipurpose *reference thesaurus *for biomedical research.

In this project, we developed a prototype, called 3D-Browser tool, which provides an interactive way to browse biomedical concepts as well as to access external information (e.g., PubMed abstracts) and HeC patient data related to these concepts. The developed prototype included the Uniprot database [34], PubMed abstracts related to the diseases studied in the project, and the HeC patient database [35]. Recently, the external web service SCAIView [36] was also integrated to provide alternative protein-disease associations mined from the literature [37].

The tool requirements were guided and evaluated by the clinicians participating in the HeC project. Moreover, the 3D browser tool was fully integrated within the workflow of other HeC related tools such as the HeC Toolbar [38], allowing selected data from the 3D-Browser to be linked with real patient data. Apart from the usability tests performed within the HeC project, we are also concerned with measuring the quality of the visualized data. As our method mainly relies on an automatic annotation system, which can produce errors and ambiguities, we must evaluate how it affects the results shown to end-users. Next sections are devoted to show use cases within the HeC project, as well as the experiments carried out to measure the quality of the generated data.

### Prototype implementation and testing

**Figure 4:**
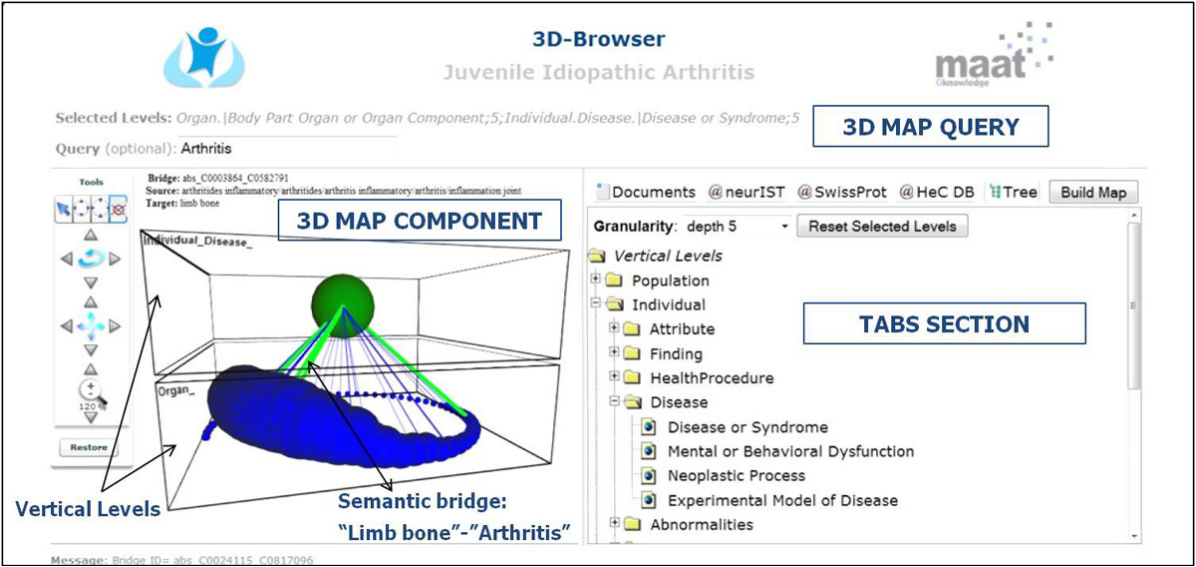
**3D Knowledge Browser snapshot with its main visual components**. This figure shows the main parts of the implemented 3D Knowledge Browser for building and exploring conceptual maps.

The current prototype of our method has been developed using AJAX (Asynchronous JavaScript and XML) technologies. Figure 4 shows an overall view of the 3D-Browser tool [19]. It consists of three main parts, namely: 1) the configuration of the conceptual map, which contains the selected vertical levels, and an optional free text query to locate concepts of interest in the conceptual map, 2) the conceptual map itself, which contains the biomedical concepts stratified in vertical levels according to the previous configuration, and 3) a series of tabs that present ranked lists of data items associated to the selected concept from the conceptual map. In the latter, each tab represents a different data collection (e.g., PubMed, Uniprot protein database, and HeC patient data). There is a special tab entitled "Tree" which contains all the possible levels that can be selected to configure and build the conceptual map. The levels are based on the UMLS semantic types [39,40] which are grouped within the corresponding HeC vertical levels as in [41]. The layers of the conceptual map can be defined by selecting levels of the "Tree" tab or through a free text query. In the second case, only the most specific concepts satisfying the query are visualized in the conceptual map.

The visual paradigm of the conceptual maps relies on the vertical integration vision proposed in HeC. That is, all the involved knowledge, data and information are organized into different disjoint vertical levels, each one representing a different perspective of the biomedical research. Figure 4 shows the stratified view of the conceptual map based on these vertical levels, in this case *Individual.Disease *and *Organ*. Within each level, biomedical concepts deemed relevant to both the clinician domain (e.g., rheumatology, cardiology and oncology) and the clinician information request are shown as *balls *in the conceptual map. The size of each ball is directly related to the concept relevance and its color indicates the operation that was performed over it, namely: green if it satisfies the free text query, red if it was expanded as a sub-concept, and blue if no action was taken on it.

**Figure 5:**
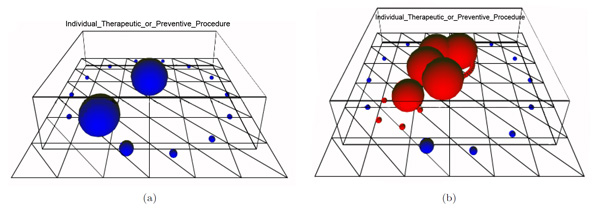
**Example of two expanded concepts: operation and implantation**. This example shows the two snapshots of a conceptual map: (a) before expanding concepts and (b) after expanding two main concepts.

*Semantic bridges *are represented as 3D lines in the conceptual map. Semantic bridges can represent either discovered co-occurrences of concepts in some target data collection or well-known relationships between concepts stated in some knowledge resource (e.g., UMLS relations). Semantic bridges can help clinicians to select the context in which the required information must hold. For example, from the conceptual map in Figure 4 we can retrieve documents or patient unique identifiers about *arthritis related to limb bones *by clicking an existing bridge between the concepts *Arthritis *and *Limb_Bone*. Finally, semantic bridges have also associated a relevance index, which depends on the correlation measure we have chosen for their definition (e.g., support, mutual information, log-likelihood ratio, etc.) The relevance of each semantic bridge is indicated by both its color (from less to more relevant: blue, green and purple) and its thickness. Thus, the semantic bridge between *Arthritis *and *Limb_Bone *can be considered as a strong connection. Another interesting feature of the conceptual maps is the ability of browsing through the taxonomical hierarchies of the biomedical concepts (e.g., UMLS hierarchy). In the example of Figure 5, the user can expand the concepts *Operation *and *Implantation *(biggest balls in Figure 5(a)). The resulting concepts are red-colored (Figure 5(b)) and represent more specific concepts like *Catheterisation*, *Surgical repair*, *Intubation*, or *Cardiovascular Operations*.

In order to manage the elements of the conceptual map a series of operations are provided in the conceptual map tools panel (see left hand-side of Figure 4). These operations are split into two categories: operations to manage the whole conceptual map (rotate, zoom and shift) and concept-related operations. The operations to manage the concept visualization involve (1) the retrieval of the objects associated to the clicked concept, (2) the expansion of the clicked concept, (3) the removal of the concepts of a level with the exception of the clicked concept, and (4) the removal of the clicked concept.

In the following paragraphs we show the functionalities of the prototype through several use examples based on some HeC clinician information requests.

#### Example 1: surgical procedures and their results in the tetralogy of Fallot domain

**Figure 6:**
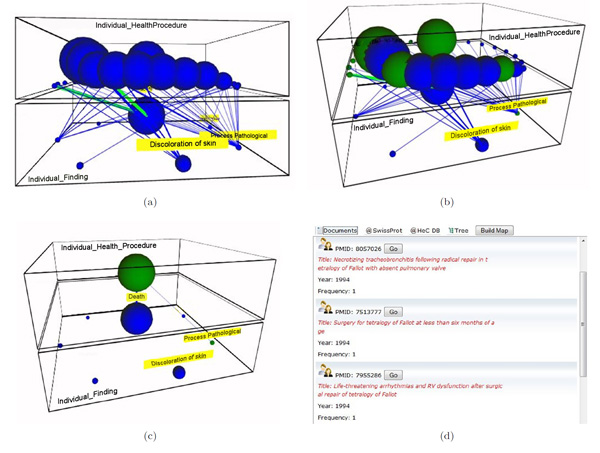
**Interesting relationships between procedures and findings in the literature**. This figure shows a sequence of actions over a conceptual map involving procedures and findings for the Tetralogy of Fallot (ToF) domain: (a) initial conceptual map built from ToF PubMed abstracts, (b) selection of nodes with the query "repair" (green balls), (c) reduction of the conceptual map to just one procedure concept, and (d) ranked list of documents retrieved for the found bridge.

Clinicians are interested in knowing the relation between the different surgical techniques reported in the literature and the findings and results that are usually correlated to them. For this purpose, a conceptual map for the semantic levels *Individual.Health_Procedures*. and *Individual.Finding *is built as shown in Figure 6(a). We can restrict the view to only repair techniques. This can be done by specifying the keyword *repair *in the query input field. The resulting conceptual map is shown in the Figure 6(b). The map can be further refined in order to focus on some concrete concept, for example *Repair Fallot Tetralogy*, just showing the concepts and bridges affected by it (see Figure 6(c)). In this case, there is just one bridge that relates the surgical technique with the outcome *Death*. Figure 6(d) shows the documents that are retrieved by clicking this bridge. Notice that these abstracts likely report death causes related to TOF repair.

**Figure 7:**
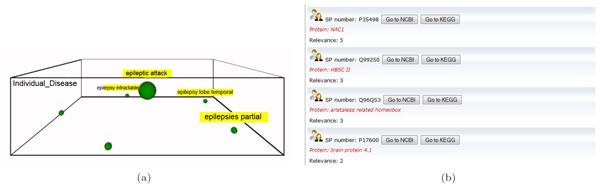
**Example of the @Swissprot tab**. This example shows the use of the @Swissprot tab. Once a conceptual map is built for the query "epilepsy" (a), we can obtain a ranked list of Uniprot protein entries related to the clicked concept (b), in this case the concept *attack epileptic*.

#### Example 2: finding potential proteins for brain tumour-related diseases

In this use case, the clinician is interested in comparing the proteins related to a disease and its subtypes. Taking the brain tumour domain, the clinician specifies the concept query *epilepsy *without selecting any vertical level. As a result, she obtains the conceptual map of Figure 7(a) which contains the concepts *attack epileptic, epilepsy intractable, epilepsy lobe temporal, epilepsy extratemporal *and *epilepsy focal*. To retrieve the proteins related to these diseases, the tab @SwissProt is selected. For example in Figure 7(b) the related proteins to *attack epileptic *are shown. The user can then get much more information about these proteins by clicking the buttons NCBI and KEGG, which jump to the corresponding pages in Entrez Gene and KEGG sites respectively. Note that, the relevance of each protein entry is calculated with the frequency of the concept and its sub-concepts in the Uniprot description of the protein.

#### Example 3: immunologic factors in juvenile idiopathic arthritis

**Figure 8:**
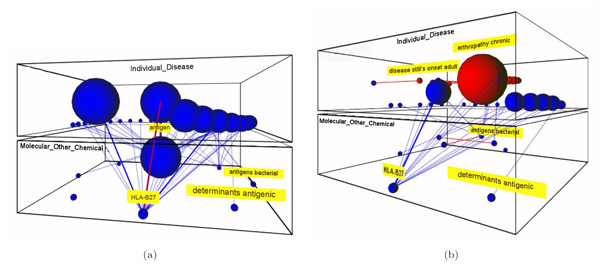
**Exploring the immunologic factors of JIA-related diseases**. This figure shows two snapshots for the conceptual map that relates diseases and immunologic factors in the JIA domain: (a) the initial conceptual map, and (b) after expanding the disease concept having the strongest relation to the HLA-B27 factor.

Juvenile idiopathic arthritis (JIA) is an autoimmune disease, that is, the immune system attacks its own cells and tissues. The cell-surface antigen HLA-B27 is well known to be associated with different kinds of JIA and it plays an important role in its classification. Moreover, male children with the HLA B27 antigen are at significantly higher risk of developing JIA. In this case, the clinician is interested in analyzing the relationships between the HLA-B27 and the different JIA subtypes, for this purpose the *Disease or Syndrome *and *Immunological factor *semantic levels are explored. As shown in the conceptual map of Figure 8, HLA-B27 plays a central role with most of the bridges associated to JIA-related diseases.

#### Example 4: location of brain tumours

**Figure 9:**
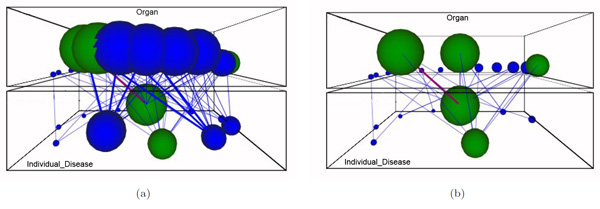
**Exploring the organ-disease bridges for cerebellum tumors**. This figure shows two snapshots for the conceptual map that relates organ and disease in the brain tumors domain: (a) the initial conceptual map with query "cerebellum", and (b) after removing non relevant concepts from the organ layer.

This example is based on the work presented in [42], which consists in retrieving patient data according to the location of the brain tumours. Figure 9(a) shows the conceptual map that relates the vertical levels *Organ *and *Disease*. Green nodes represent the relevant concepts which involve *cerebellum*. By using the node removal facility of the 3D-Browser, we can easily focus on the cerebellum related nodes (see Figure 9(b)).

### Evaluation of the quality of conceptual maps

Apart from the usability tests performed within the HeC project, we are also concerned with measuring the quality of the visualized data. As our method mainly relies on an automatic annotation system, which can produce errors and ambiguities, we must evaluate how it affects to the results shown to end-users. Data quality refers to the correctness of the system-generated multidimensional semantic spaces (MDSS) as well as the reduction achieved by the method. Wrong and ambiguous annotations can degrade the precision of the visualization by introducing misleading or noisy concepts in the conceptual maps, whereas a poor reduction of the annotation sets will introduce a lot of noise in them. Additionally, we must ensure that the reduction method captures the relevant concepts, disregarding the spurious ones.

The experiments we carried out to measure data quality have been performed over three PubMed abstract collections, one per target disease of the HeC project, namely: juvenile idiopathic arthritis (JIA), tetralogy of Fallot (ToF), and pediatric astrocytomas (AC). We use as gold-standard the MeSH indexes provided by PubMed for each abstract. We can consider that MeSH-indexes constitute a multidimensional summary of each abstract, and that we can apply the usual assessment measures for comparing our method w.r.t. the gold-standard, namely: precision (P), recall (R) and F-score. However, before applying these measures, we need to harmonize the annotations provided by our system, which refer to UMLS, and those of the gold-standard, which refer to MeSH. As MeSH is fully included in UMLS, the harmonization just consists of aligning UMLS and MeSH concepts. We consider that a UMLS concept *c*_*umls *_is aligned to a MeSH concept *c*_*mesh *_if *c*_*mesh *_≼ *c*_*mesh*_. Notice that many concepts in UMLS will be not aligned to the gold-standard, for they are not related with the MeSH taxonomy.

Table 3 presents the assessment results for these three collections. We observe that the maximum recall is around 51%, which indicates that there is a notable divergence between the gold-standard and the system generated annotations for MDSS. It is worth mentioning that many MeSH annotations regard the full version of the document, and it is likely that the annotation is not mentioned in the abstract [43]. This is why F-scores are usually low. From the results of the JIA collection we can evaluate the quality of the reduction process presented in the Methods Section. Notice that despite reducing around 55% the number of annotations, the F-score increases in both JIA and AC collections whereas in ToF there is not statistical difference. This demonstrates that the reduction method is actually keeping the relevant part of the gold-standard annotations. Notice also from this table that the size of the annotation sets of each document is similar to those of the gold-standard.

**Table 3:** Method evaluation

Domain	Docs	MeSH	MDSS	P	R	F	*L*_*MeSH*_	*L*_*mdss*_
JIA (all annot.)	7637	5096	16835	0.308	0.518	0.386	10.96	30.44
JIA (reduction)			9719	0.406	0.383	0.394		11.28

ToF (all annot.)	7669	3482	13942	0.281	0.421	0.337	10.96	26.92
ToF (reduction)			7928	0.356	0.314	0.334		9.77

AC (all annot.)	3663	3116	11898	0.221	0.477	0.301	15.37	44.8
AC (reduction)			6573	0.339	0.302	0.320	15.37	13.93

Method Evaluation. **MeSH **and **MDSS **are the number of concepts in the gold-standard and the system-generated multidimensional spaces for the three domains: JIA, ToF and AC. *P*, *R *and *F *represent the precision, recall and F-score respectively. *L*_*MeSH *_and *L*_*MDSS *_are the average number of concepts associated to each item in the gold-standard and the MDSS respectively.

In order to see the main differences between the gold-standard and MDSS representations, Table 4 reports the distribution of annotations across dimensions. From this table, we can also notice a notable divergence between both representations, specially for *Chemical*, *Drug *and *Finding *dimensions. This suggests that manual annotation has a great bias towards a few semantic types, which seem to be of special interest for PubMed users. In contrast, the concepts belonging to *Finding *are much more frequent in the abstracts than accounted by MeSH indexes.

**Table 4:** Distribution of semantic annotations

Dimension	JIA		ToF		AC	
	**MeSH**	**MDSS**	**MeSH**	**MDSS**	**MeSH**	**MDSS**

Physiology	2.0	3.9	1.9	2.3	2.8	4.7
ProteinGene	17.8	11.0	**12.1**	5.2	**24.0**	11.0
Anatomy	10.8	10.8	14.0	13.4	12.9	13.9
Drug	**9.2**	2.6	**9.0**	1.7	**8.4**	1.8
Chemical	**24.2**	7.3	**18.7**	5.2	**24.4**	6.6
Disease	23.0	15.3	25.0	19.7	22.5	17.0
HealthProcedure	13.8	19.0	17.2	20.5	13.6	16.6
Concepts	4.8	4.1	4.8	4.5	4.8	4.7
Finding	2.9	15.7	3.7	**18.6**	3.2	**14.3**
Population	6.2	6.9	6.1	6.2	4.9	6.7

This table shows the distribution of the semantic annotations generated by our method (MDSS) and the gold-standard (MeSH) for the PubMed abstract collections in the three domains: JIA, ToF and AC.

Quality of semantic bridges refers to the interestingness of the bridges generated from the multidimensional semantic spaces. Again, we use the MeSH indexes as gold-standard, and we compare the bridges generated with the gold-standard and those generated with our method. For this purpose, we have selected a few combinations of dimensions for each disease collection, which are related to the query examples of the previous section. Thus, for the JIA collection we have selected the levels *Disease *and *ProteinGene*. Tables 5 and 6 show the best scored bridges for MDSS and the gold-standard. Notice that except for three bridges, both sets are completely different. The main reason for these differences stems mainly from the different nature of the underlying annotation processes. For example, the immunologic factor IgG appears in 363 documents in the MeSH representation, whereas it only appears 12 times in MDSS. This is because the automatic semantic annotator finds more specific concepts involving IgG, like "IgG antigen", "serum IgG", and many others. Instead, MeSH-based annotation unifies all these concepts under "IgG". Additionally, as previously mentioned, some MeSH descriptors are not explicitly mentioned in the abstracts and consequently they are not regarded in the MDSS representation.

**Table 5:** MDSS-based bridges for JIA domain

Disease	ProteinGene	Score	S
anemias	erythropoietin	coh = 0.500	2
psoriasis	TNF human	coh = 0.333	2
psoriasis	fusion protein	conf = 0.333	3
periodic syndrome	TNF receptors	coh = 1.000	2
systemic onset JIA	receptors IL-6	coh = 0.114	8
rickets celiac	gluten	coh = 0.500	2
tuberculous infections	TNF blockers infliximab	conf = 0.222	2
ACLS (*)	lupus coagulation inhibitor	f1 = 0.500	2
third disease	vaccine rubella	conf = 1.000	3
diseases autoimmune	tyrosine phosphatases protein	coh = 0.500	2
syndrome laron	insulinlike growth factor	conf = 1.000	2
disorder amyloid	substance amyloid	f1 = 0.141	5
osteoarthrosis oa	proteoglycans	f1 = 0.188	3
growth failure	insulinlike growth factor	conf = 0.667	2
thyromegaly	substance amyloid	conf = 0.600	3
syndrome macrophage activation	perforin	coh = 0.571	4
syndrome macrophage activation	cyclosporine medication	f1 = 0.311	14
uveitis	factors antinuclear	conf = 0.171	19
systemic JIA	IL-1 receptor antagonist protein	conf = 0.133	2
syndrome hemophagocytic	perforin	coh = 0.286	2
eye disease cataract	factors antinuclear	conf = 0.250	15
myasthenia gravis	acetylcholine receptor	coh = 1.000	2

Best *δ*-maximum scored bridges between the *Disease *and *ProteinGene *levels for the the JIA domain, using our reduction method (MDSS). Interestingness measures used are: cohesion (coh), confidence (conf) and f-measure (f1). The column *S *indicates the number of documents supporting each bridge.

**Table 6:** MeSH-based bridges for the JIA domain

Disease	ProteinGene	Score	S
systemic rheumatoid arthritis	rheumatoid factor	coh = 0.290	105
systemic rheumatoid arthritis	IgG	coh = 0.262	95
essential anemia	ferritins	coh = 0.150	3
essential anemia	hemoglobins	coh = 0.150	3
recurrent polyserositis	proteins cytoskeletal	coh = 1.000	3
hyperhomocysteinemias	l-homocysteine	coh = 1.000	2
spondylitis rheumatoid	leukocyte antigens human	coh = 0.219	49
stills disease adult-onset	receptors IL-6	coh = 0.158	3
system lupus erythematosus	autoimmune antibody	coh = 0.206	29
disorder amyloid	SAA protein	coh = 0.828	24
disorder amyloid	substance amyloid	coh = 0.667	14
third disease	vaccine rubella	coh = 0.500	2
iron-deficiency anemias	transferrin receptor	coh = 0.750	3
infection	interferon	coh = 0.286	2
thyromegaly	SAA protein	coh = 0.500	2
myasthenia gravis	acetylcholine receptor	coh = 1.000	2
hyperimmunoglobulinemias	IgD	coh = 0.375	3
uveitis	factors antinuclear	coh = 0.184	49
thyroid insufficiency	thyroxine	coh = 1.000	2
thyroid insufficiency	thyroglobulins	f1 = 0.500	2
rickets celiac	gliadin	coh = 0.800	4
castleman's disease	receptors IL-6	coh = 0.833	5
castleman's disease	IL-6	coh = 0.833	5

Best *δ*-maximum scored bridges between the *Disease *and *ProteinGene *levels for the the JIA domain, using the gold-standard (MeSH). Interestingness measures used are: cohesion (coh), confidence (conf) and f-measure (f1). The column *S *indicates the number of documents supporting each bridge.

For the ToF collection, we have selected the levels *Disease *and *HealthProcedure*, restricting them to the semantic types *CongenitalAbonormality *and *Therapy *respectively. Tables 7 and 8 show the best scored bridges for the MDSS and MeSH-based representations respectively. Notice that in this case, bridges indicate relations between abnormalities and surgical methods applied to them. For the MeSH representation, bridges always refer to "surgical procedures heart", but not to any specific technique. This is again due to the MeSH-based manual annotation of abstracts, which systematically selects this concept when an abstract talks about heart surgical procedures.

**Table 7:** MDSS-based bridges for the ToF domain

Disease.CongenitalAbnormality	HealthProcedure.Therapy	Score	S
stricture pulmonary artery congenital	procedure fontan	coh = 0.250	2
stricture pulmonary artery congenital	stent s biliary	coh = 0.250	3
right ventricular dilatation	replacement pulmonary valve	conf = 0.231	3
major aortopulmonary collateral artery	therapy embolization	coh = 0.200	3
tetralogy fallots	surgical repairs	coh = 0.611	251
tetralogy fallots	surgical treatment	coh = 0.517	733
congenital pulmonary artery aneurysm	pericardial shunt operation	conf = 0.154	2
congenital pulmonary artery aneurysm	arteriovenous shunt procedure	coh = 0.154	2
single coronary artery	anomalous coronary artery graft treatment	coh = 0.167	3
syndrome alagille	transplant liver	coh = 1.000	2
peripheral pulmonary artery stenosis	stent s biliary	coh = 0.333	2
cross syndrome	reperfusions	coh = 0.400	2
infantile lobar emphysema	lobectomy	coh = 0.500	2
ventricular septal defect spontaneous closure	surgical closure	coh = 0.125	5

Best *δ*-maximum scored bridges between the *CongenitalAbnormality *and *Therapy *levels for the the ToF domain, using our reduction method (MDSS). Interestingness measures used are: cohesion (coh), confidence (conf) and f-measure (f1). The column *S *indicates the number of documents supporting each bridge.

**Table 8:** MeSH-based bridges for the ToF domain

Disease.CongenitalAbnormality	HealthProcedure.Therapy	Score	S
tetralogy fallots	surgical procedures heart	coh = 0.837	498
vessels transposition great	surgical procedures heart	coh = 0.106	63
malformation heart	surgical procedures heart	coh = 0.329	196
vsd ventricular septal defect	surgical procedures heart	coh = 0.200	119
septal defects atrial	surgical procedures heart	coh = 0.108	61

Best *δ*-maximum scored bridges between the *CongenitalAbnormality *and *Therapy *levels for the the ToF domain, using the gold-standard (MeSH). Interestingness measures used are: cohesion (coh), confidence (conf) and f-measure (f1). The column *S *indicates the number of documents supporting each bridge.

Finally, for the AC collection we have selected the dimensions *Anatomy*, restricted to cells, and *Disease *restricted to neoplastic processes. Tables 9 and 10 show the generated bridges. In this case, the MDSS method obtains a much richer set of bridges than those generated from the gold-standard.

**Table 9:** MDSS-based *δ*-maximum scored bridges for the AC domain

Anatomy.Cell	Disease.NeoplasticProcess	Score	S
tumor cell	pleomorphic xanthoastrocytoma	coh = 0.125	7
human cell line	small-cell glioblastoma	coh = 1.000	2
tumour cells	g-cell tumor	coh = 0.333	2
oligodendroglial cell	oligodendrogliomas	conf = 0.190	4
neurons	dysplasias	coh = 1.000	2
multinucleate giant cell	glioblastomas giant cell	coh = 1.000	2
multinucleate giant cell	subependymal giant cell astrocytoma	coh = 0.130	3
multinucleate giant cell	tuberous sclerosis syndrome	conf = 0.217	5
multinucleate giant cell	pleomorphic xanthoastrocytoma	conf = 0.174	4
spindle cell	subependymal giant cell astrocytoma	coh = 0.400	2
tumour cell lines	solid tumour childhood	coh = 0.667	2

Best *δ*-maximum scored bridges between the *Cell *and *NeoplasticProcess *levels for the the AC domain, using our reduction method (MDSS). Interestingness measures used are: cohesion (coh), confidence (conf) and f-measure (f1). The column *S *indicates the number of documents supporting each bridge.

**Table 10:** MeSH-based *δ*-maximum scored bridges for the AC domain

Anatomy.Cell	Disease.NeoplasticProcess	Score	S
neurons	gangliogliomas	coh = 0.160	4
cultured cells	melanoma syndrome	coh = 0.158	3
t-lymphocytes	malignant adenomas	coh = 0.286	2
tumour cell lines	neoplasms experimental	coh = 0.500	2

Best *δ*-maximum scored bridges between the *Cell *and *NeoplasticProcess *levels for the the AC domain, using the gold-standard (MeSH). Interestingness measures used are: cohesion (coh), confidence (conf) and f-measure (f1). The column *S *indicates the number of documents supporting each bridge.

Concluding, our method generates interesting bridges comparable in quality to those generated from the gold-standard. It is worth mentioning that we have found very few errors due to the semantic annotation system. An example of error is shown in Table 5, where ACLS is not a disease. Finally, due to the significant divergence present in the MDSS and the gold-standard representations, bridges derived from them can vary greatly. Future work must pay attention to the impact of the used annotation method in both the resulting multidimensional space and its generated bridges.

## Conclusions

Current knowledge resources and semantic-aware technology make possible the integration of biomedical resources. Such an integration is achieved through semantic annotation of the intended biomedical resources. This paper shows how these annotations can be exploited for integration, exploration, and analysis tasks.

The presented approach relies on multidimensional semantic spaces and OLAP-style operators, which has been shown suitable for browsing biomedical information. We also show that the same knowledge resources that support the semantic annotations (i.e., thesauri and domain ontologies) provide the necessary elements to build the taxonomical dimensions that facilitate the exploration of the semantic spaces. The viability of the approach is finally demonstrated with the developed prototype (3D-Browser), which has been tested over a real scenario.

As for the quality of the generated semantic spaces, we show that the conceptual representations of our approach are partially complementary to the representation given by MeSH descriptors. The normalization process defined to accommodate the semantic annotations into the given dimensions does not suffer from quality loss. The quality of discovered bridges is usually similar or, in some cases, better than those derived from the MeSH descriptors.

As future work, it would be interesting to investigate probabilistic translation methods [44] for different conceptual representations, so that the quality of the semantic annotations can be further improved. For example, with these methods, some hidden concepts in the abstract that are captured by MeSH descriptions could be discovered by other annotation systems. Other future work will be focused on the discovery of interesting bridges using association rules algorithms. Recently, we have investigated in [45] the generation of rules from semantic annotations derived from patient record databases. These rules could be included in the proposed conceptual maps for exploring them as well as for comparing them to existing bridges. Finally, we will investigate how to include in our approach those semantic relationships that are being extracted from the literature, as those obtained with the DIDO tool [46].

## List of abbreviations

AC: Astrocytoma; AJAX: Asynchronous JavaScript and XML; CALBC: Collaborative Annotation of a Large Biomedical Corpus; CR: Concept Retrieval; DR: Data resources; GO: Gene Ontology; HeC: Health-e-Child; JIA: Juvenile Idiopathic Arthritis; KEGG: Kyoto Encyclopedia of Genes and Genomes; KR: Knowledge Resources; LBD: Literature Based Discovery; MDSS: Multidimensional Semantic Spaces; MeSH: Medical Subject Headings; NCBI: National Center for Biotechnology Information; OLAP: OnLine Analytical Processing; OMIM: On-line Mendelian Inheritance in Man; OWL: Ontology Web Language; RDF: Resource Description Framework; TOF: Tetralogy of Fallot; UMLS: Unified Medical Language System; XML: eXtended Mark-up Language.

## Competing interests

The authors declare that they have no competing interests.

## Authors' contributions

RB designed the 3D-Browser and carried out its implementation (client-side) as well as the normalization methods. EJR developed the HeC use cases and designed the experiments related to them. VN implemented and adapted the ontology indexing scheme over which most of the browser operations are performed in the back-end. All authors drafted, read and approved the final manuscript.
